# Two New N-Oxide Alkaloids from *Stemona cochinchinensis*

**DOI:** 10.3390/molecules191220257

**Published:** 2014-12-03

**Authors:** Ligen Lin, Han Bao, Anqi Wang, Chunping Tang, Pham-Huu Dien, Yang Ye

**Affiliations:** 1 State Key Laboratory of Quality Research in Chinese Medicine, Institute of Chinese Medical Sciences, University of Macau, Macao 999078, China; E-Mails: mb45828@umac.mo (H.B.); yb37510@umac.mo (A.W.); 2 State Key Laboratory of Drug Research, Shanghai Institute of Materia Medica, Chinese Academy of Sciences, Shanghai 201203, China; E-Mail: tangcp_sh@163.com; 3 Department of Organic Chemistry, Faculty of Chemistry, Hanoi National University of Education, Hanoi 100000, Vietnam; E-Mail: dienph@hnue.edu.vn

**Keywords:** *Stemona cochinchinensis*, Stemonaceae, N-oxide alkaloids, ^1^H- and ^13^C-NMR, anti-tussive activity

## Abstract

Two new N-oxide alkaloids with pyrrolo[1,2-α]azepine skeleton, namely isoneostemocochinine-N-oxide (**1**) and neostemocochinine-N-oxide (**2**), as well as three known alkaloids with pyrido[1,2-α]azepine skeletons, were isolated and identified from the roots of *Stemona cochinchinensis* (Stemonaceae). The structures of these compounds were elucidated by 1D- and 2D-NMR spectra and other spectroscopic studies. Additionally, the ^1^H- and ^13^C-NMR characteristic of N-oxide *Stemona* alkaloids was summarized. Stemokerrin showed potent anti-tussive activity on citric acid-induced guinea pig model.

## 1. Introduction

**Figure 1:**
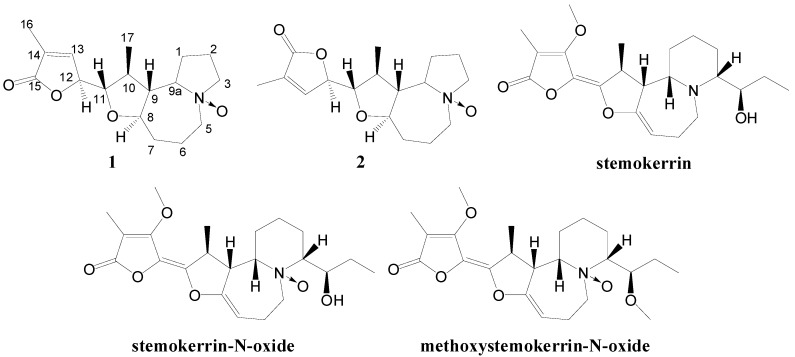
Structures of the isolated alkaloids.

The genus *Stemona* (Stemonaceae) has been used as a source of insecticides and antitussive remedies in China, Japan and Southeastern Asian countries for a long time [1,2]. Till now, about 140 alkaloids have been isolated from the *Stemona* genus and structurally identified, most of which share the common pyrrolo[1,2-α]azepine nucleus, while several contain a pyrido[1,2-α]azepine skeleton [3,4,5,6,7,8,9]. *Stemona cochinchinensis* Gagnep. is one of the three endemic *Stemona* species in Vietnam. We and others have reported twelve alkaloids with a basic pyrrolo[1,2-α]azepine nucleus, and six alkaloids with pyrido[1,2-α]azepine skeletons from this species [4,6,8,10]. In our preliminary investigation on this plant, we also reported three bisbenzopyrans [10]. Herein a further investigation of the minor alkaloidal constituents from the title plant was carried out. We describe the isolation and structural elucidation of two new N-oxide alkaloids with pyrrolo[1,2-α]azepine skeletons, namely bisoneostemocochinine-N-oxide (**1**) and neostemocochinine-N-oxide (**2**) (Figure 1). Three known alkaloids were also obtained and identified as stemokerrin [4], stemokerrin-N-oxide [7] and methoxystemokerrin-N-oxide [4]. Their structures were elucidated by 1D- and 2D-NMR analysis together with other spectroscopic studies. Furthermore, the N-oxide *Stemona* alkaloids were reviewed and their NMR spectral characteristics were discussed. The major alkaloid, stemokerrin, was chosen to test for anti-tussive activity using a citric acid-induced guinea pig model.

## 2. Results and Discussion

**Figure 2:**
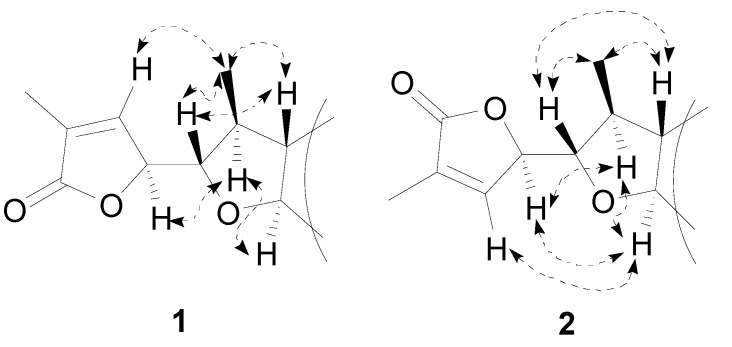
Key NOE correlations of compounds **1** and **2**.

Isoneostemocochinine-N-oxide (**1**) was obtained as a yellow amorphous powder. The HR-ESI-MS suggested the molecular formula C_17_H_2__5_NO_4_ (*m*/*z* 330.1672 [M+Na]^+^; calc. 330.1681). The strong and sharp IR band at 1,775 cm^−1^ indicated the existence of a *γ*-lactone. The ^1^H-NMR spectrum (Table 1) displayed resonances of two methyl groups [δ_H_ 1.15 (3H, d, *J* = 6.1 Hz, CH_3_-17) and 1.92 (3H, d, *J* = 1.7 Hz, CH_3_-16)], an olefinic proton [δ_H_ 6.98 (q, *J* = 1.7 Hz, H-13)], and three low-field protons [δ_H_ 3.72 (m, H-11), 3.94 (m, H-8) and 4.88 (m, H-12)]. The ^13^C-NMR spectrum of compound **1** showed 17 resonances, which were classified into one *sp^2^* carbonyl carbon, two *sp^2^* olefinic carbons, six *sp^3^* tertiary carbons, six *sp^3^* secondary carbons and two *sp^3^* methyl carbons (Table 1). The ^1^H- and ^13^C-NMR spectra of compound **1** strongly resembled those of isoneostemocochinine [8]. The molecular weight of compound **1** was 16 Da more than that of isoneostemocochinine, which suggested the possible presence of an N-oxide moiety in the molecule of **1**. This was further supported by the down-field shifts of H-3 (3.48, m), H-5α (3.95, m), H-5β (3.60, m), H-9a (3.89, m), C-3 (70.2), C-5 (66.0) and C-9a (80.6) in comparison with those corresponding NMR data of isoneostemocochinine (Table 2). Therefore the planar structure of compound **1** was determined. The relative configuration of **1** was inferred by the ROESY experiment (broken arrows in Figure 2). In all the stemoamide-group alkaloids, H-9 is β-oriented [3]. The correlations of H-11/H-9, CH_3_-17/H-9 and CH_3_-17/H-11 indicated that H-11 and CH_3_-17 were both β-oriented. The relative configuration of C-12 was determined to be rel-S by the ROESY correlations of H-12/H-10 and H-13/CH_3_-17. Thus the full structure of **1** was established and the detailed assignments of the ^1^H- and ^13^C-NMR resonances were shown in Table 1.

**Table 1:** ^1^H-NMR and ^13^C-NMR data for compounds **1** and **2** (CDCl_3_).

	1		2	
	δ_H_, *J* (Hz)	δ_C_	δ_H_, *J* (Hz)	δ_C_
1	1.82 m	20.5	1.81 m	20.6
1	1.57 m		1.57 m	
2	1.91 m	24.6	1.91 m	24.8
2	1.37 m		1.37 m	
3	3.48 m (2H)	70.2	3.48 m (2H)	70.4
5α	3.95 m	66.0	3.95 m	66.0
5β	3.60 m		3.62 m	
6	1.54 m	18.8	1.54 m	18.9
6	1.37 m		1.37 m	
7	2.06 m	32.4	2.06 m	32.1
7	1.27 m		1.27 m	
8	3.94 m	83.0	4.05 m	82.8
9	2.11 m	48.7	2.17 m	49.0
9a	3.89 m	80.6	3.89 m	80.5
10	2.22 m	40.2	2.20 m	39.6
11	3.72 m	84.6	3.82 m	84.6
12	4.88 m	80.5	4.93 m	80.2
13	6.98 q (1.7)	145.8	7.07 q (1.7)	145.8
14		131.1		131.3
15		174.2		173.9
16	1.92 d (1.7)	10.8	1.92 d (1.7)	10.7
17	1.15 d (6.1)	14.9	1.09 d (6.1)	16.3

**Table 2:** Comparison of NMR data of N-oxide *Stemona* alkaloids and their precursors.

Compound	δ_H_						δ_C_						Ref
	H-3	Δ	H-5	Δ	H-9a	Δ	C-3	Δ	C-5	Δ	C-9a	Δ	
isoneostemocochinine N-oxide	3.48 (2H)	0.20	3.95/3.60	0.47/0.64	3.89	0.25	70.2	18.2	66.0	16.2	80.6	19.9	[8]
isoneostemocochinine	3.28 (2H)		3.48/2.96		3.64		52.0		49.8		60.7		
neostemocochinine N-oxide	3.48 (2H)	0.20	3.95/3.62	0.46/0.64	3.89	0.25	70.4	18.3	66.0	16.1	80.5	19.8	[8]
neostemocochinine	3.28 (2H)		3.49/2.98		3.64		52.1		49.9		60.7		
stemaphylline N-oxide	3.57 (2H)	0.56/1.06	3.33 (2H)	0.39/0.82	4.65	1.72	71.0	16.7	67.2	14.9	81.6	16.8	[11]
stemaphylline	3.01/2.51		2.94/2.51		2.93		54.3		52.3		64.8		[11]
stenine A	3.79/3.69	0.14/0.22	3.87/3.54	0.74/0.58	3.69	1.62	72.2	19.5	67.7	15.4	84.3	19.0	[12]
stenine B	3.65/3.47		3.13/2.96		2.07		52.7		52.3		65.3		[12]
N-oxytuberostemonine	3.67	0.24	3.78/3.52	0.31/0.85	4.01	0.94	77.5	12.5	69.7	21.6	88.9	25.3	[13]
tuberostemonine	3.43		3.47/2.67		3.07		65.0		48.1		63.6		[14]
	**H-4**	**Δ**	**H-6**	**Δ**	**H-10a**	**Δ**	**C-4**	**Δ**	**C-6**	**Δ**	**C-10a**	**Δ**	****
stemocurtisine N-oxide	3.44/3.29	0.42/0.42	4.12/3.01	0.74/0.05	3.66	0.22	69.6	16.0	66.4	13.4	82.3	20.3	[9]
stemocurtisine	3.02/2.87		3.38/2.96		3.44		53.6		53.0		62.0		[15]
oxystemokerrine N-oxide	3.31	0.72	4.04/3.55	0.68/0.49	3.93	0.43	79.4	13.6	61.9	17.7	85.4	19.0	[4]
oxystemokerrine	2.59		3.36/3.06		3.50		65.8		44.2		66.4		[4]
stemokerrine N-oxide	3.22	0.62	3.46/3.42	0/76/0.86	3.22	0.39	81.9	12.0	54.1	14.4	78.3	15.9	[7]
methoxystemokerrine N-oxide	3.26	0.66	3.36/2/63	0.66/0.07	3.21	0.38	84.3	14.4	56.2	16.5	78.5	16.1	[4]
stemokerrine	2.60		2.70/2.56		2.83		69.9		39.7		62.4		[4]

Neostemocochinine-N-oxide (**2**) was obtained as a yellow amorphous powder. The molecular formula was determined by the HR-ESI-MS to be C_17_H_2__5_NO_4_ (*m*/*z* 330.1689 [M+Na]^+^; calc. 330.1681), the same as that of **1**. The presence of a *γ*-lactone was indicated by the strong and sharp absorption band at 1770 cm^−1^ in the IR spectrum. The ^1^H- and ^13^C-NMR data of **2** (Table 1) were similar to those of neostemocochinine [8]. The molecular weight difference and down-field shifts of H-3, H-5, H-9a, C-3, C-5 and C-9a in ^1^H- and ^13^C-NMR (Table 2) suggested that compound **2** was an N-oxide of neostemocochinine. A careful analysis of the spectroscopic data resulted in the conclusion that compounds **2** and **1** shared the same planar structure. The major differences of their NMR data involved the chemical shifts of H-11, H-12, H-13 and C-10 (Table 1), suggesting **2** was a stereoisomer of **1**. The relative configuration of **2** was disclosed by the ROESY spectrum (broken arrows in Figure 2). The correlations of H-11/H-9 and H-11/CH_3_-17 revealed that H-9, H-11 and CH_3_-17 were β-oriented. The correlations of H-12/H-10, H-12/H-8 and H-13/H-8 suggested an rel-*R*-configuration of C-12. The stereochemistry of C-11 and C-12 was consistent with that of neostemocochinine [8].

**Figure 3:**
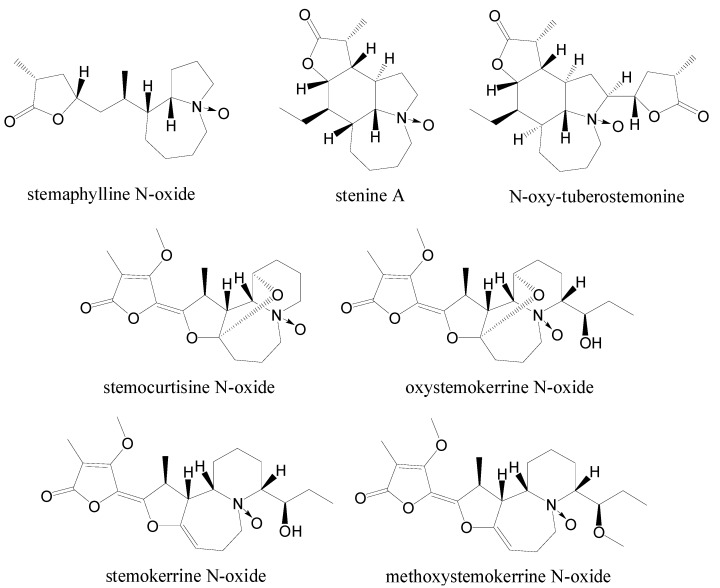
N-oxide *Stemona* alkaloids.

The natural occurring N-oxide *Stemona* alkaloids are rare, and till now only nine were isolated and identified including compounds **1** and **2** (Figure 1 and Figure 3 ).

Five of them, including isoneostemocochinine N-oxide (**1**), neostemocochinine N-oxide (**2**), stemaphylline N-oxide [11], stenine A [12] and N-oxy-tuberostemonine [13], contain pyrrolo[1,2-α]azepine skeletons. The other four including stemocurtisine N-oxide [9], oxystemokerrine N-oxide [4], stemokerrine N-oxide [7] and methoxystemokerrine N-oxide [4] contain pyrido[1,2-α]azepine skeletons. Most of the N-oxide *Stemona* alkaloids were identified from *Stemona* species collected from Southeast Asia, including *S. cochinchinensis*, *S. saxorum*, *S. curtisii*, *S. kerrii* and *S. aphylla*. It might be due to warm and moist environment in this area.

As the electronegativity of oxygen atom is stronger than that of nitrogen, the electron cloud in nitrogen oxides is attracted toward the oxygen end. The deshielding effect results in the down-field shifts of hydrogens and carbons near the nitrogen atom in NMR spectra. Regarding N-oxide *Stemona* alkaloids, the chemical shifts of H-3 (H-4), H-5 (H-6) and H-9a (H-10a) are 0.14 to 1.72 ppm more than their corresponding signals in precursors (Table 2). Additionally, the signals of C-3 (C-4), C-5 (C-6) and C-9a (C-10a) in N-oxide *Stemona* alkaloids are down-field shift about 12.0 to 25.3 ppm, compared with their corresponding signals in precursor alkaloids (Table 2). These data could help to elucidate the structures of N-oxide *Stemona* alkaloids.

The major alkaloid, stemokerrin, was tested for anti-tussive activity in the citric acid-induced guinea pig cough model. Stemokerrin showed significant anti-tussive activity, and about 62% cough inhibition was achieved at a single intraperitoneal (ip) dose of 70 mg/kg. Comparing the results from our previous study on stemoninine (about 43% cough inhibition at a single ip dose of 75 mg/kg) [16], stemokerrin exhibited markedly higher cough suppression potency. The current study thus has disclosed the first pyrido[1,2-α]azepine skeleton alkaloid with potent anti-tussive activity, which might contribute to the development of a new cough inhibition therapy.

## 3. Experimental Section

### 3.1. General Information

Column chromatography (CC) was performed with commercial silica gel (QingDao Marine Chemical Industrials, Qingdao, China) and Sephadex LH-20 (GE Healthcare, Wauwatosa, WI, USA). TLC was performed with precoated silica gel GF254 plates (Yantai Chemical Industrials, Yantai, China). The optical rotations were recorded using a Perkin-Elmer 341 polarimeter (PerkinElmer, Waltham, MA, USA). The UV spectra were measured on a Hewlett-Packard 8452A diode array spectrophotometer (Agilent Technologies, Palo Alto, CA, USA). The IR spectra were obtained as KBr pellets on a Nicolet Magna FT-IR 750 spectrophotometer (Thermo, Pittsburgh, PA, USA). The NMR spectra were recorded on Bruker AM-400 (Bruker, Bremen, Germany), using TMS as internal standard, assignments supported by ^1^H, ^1^H-COSY, HSQC, ROESY and HMBC experiments. EI-MS spectra: Finnigan MAT-95 mass spectrometer (Thermo). ESI-MS and HR-ESI-MS spectra were measured with a Bruker MicroToF LC-MS-MS mass spectrometer (Bruker).

### 3.2. HPLC Conditions

Analytical HPLC was performed on a Waters 2690 instrument with a 996 Photodiode Array Detector (PAD) and an Alltech ELSD 2000 detector. Chromatographic separation was carried out on an XTerra RP18 column (4.6 × 250 mm, 5 μm, Waters, Milford, MA, USA), using a gradient solvent system comprised of H_2_O (A) and CH_3_CN (B) containing 0.1% ammonia, at a flow rate of 1.0 mL/min. Temperature for the ELSD drift tube was set at 105 °C, and the air flow was 3.2 L/min. Preparative HPLC was performed on a Varian SD1 instrument with 320 single wave detector. Chromatographic separation was carried out on a C18 column (220 × 25 mm, 10 μm, Merck, Darmstadt, Germany), using a gradient solvent system comprised of H_2_O (A) and CH_3_CN (B) containing 0.1% ammonia, at a flow rate of 15 mL/min.

### 3.3. Plant Material

The roots of *S. cochinchinensis* (1.52 kg) were collected in Sonla province of Northern Vietnam in April 2002 by Nguyen Duc Thinh, Director of Sonla State Farm and identified by Vu Ngoc Chuyen, Hanoi University of Pharmacy.

### 3.4. Extraction and Isolation

The crude alkaloids were extracted as described previously [8]. The crude alkaloids (15 g) were subjected to column chromatography over silica gel and eluted with petroleum ether–acetone gradients from 9:1 to 1:1, acetone and then methanol, to yield 12 fractions. Fraction 4 (2.115 g) was subjected to column chromatography over silica gel eluting with petroleum ether–acetone (9:1) to obtain stemokerrin (201 mg). Fraction 7 (1.546 g) was separated by column chromatography over silica gel with petroleum ether–acetone (4:1) and then purified with preparative HPLC (CH_3_CN–H_2_O from 40% to 55% in 0–60 min and then from 55% to 70% in 60–180 min), affording stemokerrin-N-oxide (34 mg) and methoxystemokerrin-N-oxide (13 mg). Fraction 8 (860 mg) was subjected to column chromatography over silica gel (petroleum ether–acetone 4:1) and Sephadex LH-20 (chloroform–methanol (1:1) repeatedly to give five subfractions (1–5). Subfraction 4 was further separated by preparative HPLC (CH_3_CN–H_2_O 30:70) to yield isoneostemocochinine N-oxide (**1**, 8 mg) and neostemocochinine N-oxide (**2**, 6 mg).

*Isoneostemocochinine* *N-oxide* (**1**). Yellow amorphous powder; ${\left[\alpha \right]}_{D}^{20}$ −54 (*c* = 0.10, CHCl_3_); UV (MeOH) λ_max_ 239.2 (2.35); IR (KBr) ν_max_ 1775, 1456, 1213, 1094, 1045, 752; ^1^H- and ^13^C-NMR see Table 1; positive ESI-MS *m*/*z* 308.1 [M+1]^+^, 615.1 [2M+1]^+^; EIMS *m*/*z* 291, 289, 271, 256, 194, 192, 177, 164, 162, 134, 120, 111, 96, 84, 70; HR-ESI-MS *m*/*z* [M+Na]^+^ 330.1672 (calcd. for C_17_H_2__5_NO_4_Na, 330.1681).

*Neostemocochinine* *N-oxide* (**2**). Yellow amorphous powder; ${\left[\alpha \right]}_{D}^{20}$ −12 (*c* = 0.10, CHCl_3_); UV (MeOH) λ_max_ 239.1 (2.35); IR (KBr) ν_max_ 1770, 1439, 1161, 1082, 1051, 754; ^1^H- and ^13^C-NMR see Table 1; positive ESI-MS *m*/*z* 308.1 [M+1]^+^, 615.2 [2M+1]^+^; HR-ESI-MS *m*/*z* [M+Na]^+^ 330.1689 (cacld. for C_17_H_2__5_NO_4_Na, 330.1681).

### 3.5. Anti-Tussive Activity of Stemokerrin

The citric acid-induced guinea pig cough model was used in this study, as described in our previous study [16]. Briefly, unrestrained, conscious Dunkin-Hartley guinea pigs of both sexes (300–350 g) were randomly divided into groups with at least five animals in each group. Stemokerrin (dose 70 mg/kg) was given to the guinea pigs via a single ip injection. The treated animal was individually placed into a transparent Perspex airtight chamber. At 30 min after treatment, each animal was exposed to 0.5 M citric acid aerosols for 8 min with a flow rate of 0.5 mL/min. During the aerosol exposure, the animal was continuously monitored, and cough sounds were recorded via a microphone connected to a personal computer and analyzed by Cool Edit 2000 software (Syntrillium, Phoenix, AZ, USA). Cough episodes were determined. The antitussive activity of codeine phosphate as the positive control and response of the vehicle control (Tween 80 in saline (5:95, *v*/*v*)) were also tested in parallel studies. Antitussive activity was evaluated and expressed as the percentage of cough inhibition based on the comparison of numbers of cough episodes recorded in the alkaloid-treated group with the corresponding vehicle control group.

## 4. Conclusions

In the present study, two new N-oxide *Stemona* alkaloids named isoneostemocochinine-N-oxide and neostemocochinine-N-oxide were isolated from the roots of *S. cochinchinensis*. The NMR characteristics of N-oxide *Stemona* alkaloids was also summarized, which might help to the structural elucidation of novel alkaloids. The present study also discovered the first pyrido[1,2-α]azepine skeleton alkaloid, stemokerrin, with potent anti-tussive activity.
